# Analysis of similarities and differences of accessions belonging to Prunus domestica L. and P. insititia L. using endocarp dimensions and shape variations

**DOI:** 10.18699/vjgb-25-06

**Published:** 2025-02

**Authors:** T. Milošević, N. Milošević

**Affiliations:** Department of Fruit Growing and Viticulture, Faculty of Agronomy in Čačak, University of Kragujevac, Čačak, Republic of Serbia; Depаrtment of Pomology and Fruit Breeding, Fruit Research Institute, Čačak, Republic of Serbia

**Keywords:** endocarp, European and Damson plums, morphological properties, size, shape indexes, эндокарпий, слива европейская и чернослива, морфологические свойства, размер, индексы формы

## Abstract

The endocarp or stone is the most stable morphological feature of the genus Prunus. However, the identification of plum types, groups and/or genotypes based on endocarp is complicated because of a wide range of variation and morphological transitional states. From this point of view, knowledge on the degree of variability within and between plum species or cultivars is a sine qua non for taxonomists and also for pomologists. In this study, different endocarp morphological traits, such as SW, linear dimensions (L, W and T), Da, Dg, S, V and shape indexes (φ, SI, E, RS, RO, DE and PI) were determined using analysis of variance and multivariate analysis (correlations and PCA). Results showed significant differences among accessions for all properties evaluated but with high overlaps in values. In most cases, the examined parameters were positively or negatively correlated with each other, indicating developmental relationships between them. Indeed, positive correlations were recorded for most variables, especially related to SW and endocarp linear dimensions. These results showed that the above properties could be a powerful indicator for selecting adequate endocarp size and shape in accessions, which may be used in taxonomic analysis. With an account of these correlations, PCA was employed to correctly estimate the endocarp size and shape and distribution, segregation and dispersion of accessions. All linear measurements and index values showed a normal or low variability at the individual level in most cases, with the exception of SW, V and PI in both European and Damson plums and S in Damson plums. Of the 15 examined parameters, European plum had significantly higher SW, L, T, Da, Dg, S, E, RO and PI values than Damson plum. In contrast, Damson plum had higher SI, RS and DE values, while W, V and φ were similar.

## Abstract (ru)

Эндокарпий, или косточка, является наиболее стабильной морфологической особенностью рода Prunus. Однако идентификация типов, групп и/или генотипов сливы на основе эндокарпия осложнена из-за широкого спектра вариаций и морфологических переходных состояний. С этой точки зрения знание степени изменчивости внутри и между видами или сортами сливы является непременным условием для таксономистов и помологов. В нашем исследовании различные морфологические признаки эндокарпия, такие как SW, линейные размеры (L, W и T), Da, Dg, S, V и индексы формы (φ, SI, E, RS, RO, DE и PI), были определены с помощью дисперсионного анализа и многомерного анализа (корреляции и PCA). В результате обнаружены значительные различия между образцами по всем оцененным свойствам, но с высоким перекрытием значений. В большинстве случаев исследованные параметры положительно или отрицательно коррелировали друг с другом, что указывало на связи развития между ними. Действительно, положительные корреляции были зарегистрированы для большинства переменных, особенно связанных с SW и линейными размерами эндокарпия. Эти результаты показали, что вышеперечисленные свойства могут служить мощным индикатором для выбора адекватного размера и формы эндокарпия образцов, которые могут быть использованы в таксономическом анализе. Выявленные корреляции были использованы для проведения анализа методом главных компонент (PCA), что позволило правильно оценить не только размеры и форму эндокарпия, но и диапазоны варьирования признаков, включая дисперсию, а также распределение образцов по группам. Все линейные измерения и значения индекса показали нормальную или низкую изменчивость на индивидуальном уровне в большинстве случаев, за исключением SW, V и PI у европейской сливы и сливы Damson и S у сливы Damson. Из 15 исследованных параметров для европейской сливы были получены значительно более высокие значения SW, L, T, Da, Dg, S, E, RO и PI по сравнению со сливой Damson. Напротив, слива Damson отличалась более высокими значениями SI, RS и DE, тогда как значения W, V и φ были схожими.

## Introduction

Prunus spp. has been grown throughout the world for centuries. Among commercial species, the plum is the most commonly cultivated for its fruits (Milošević T., Milošević N., 2018). The genus originates from five centers in general: Europe for Prunus domestica L. (European plum), Western Asia for P. insititia L. (Damson plum), Western and Central Asia for P. cerasifera Ehrh. (cherry plum), North America for P. americana Marsh. (American plum) and China for P. salicina Lindl. (Japanese plum) (Watkins, 1976). Among them, genotypes belonging to P. domestica L. and P. salicina Lindl. are the most important. The evaluation of plum diversity may be essential; for example, for on-farm conservation schemes, utilization of genetic resources for sustainable agriculture and future breeding programs (Ropelewska, 2022). Also, cultivar differentiation is important for farming due to adaptation to climatic conditions and disease resistance, especially under global warming conditions (Milošević T., Milošević N., 2018).

Serbia is a small country on the Balkan Peninsula, but a major world producer of plums, as it ranks third or fourth in the world behind China, USA or Romania (FAOSTAT, 2024, https://www.fao.org/faostat/en/#data/QCL). Apart from a large number of commercial cultivars, Serbia is known for growing autochthonous (primitive, folk) genotypes mostly originating from P. domestica L. (European plum) and P. insititia L. (Damson plum) (Milošević T., Milošević N., 2012). Their fruits are mainly used for the production of a traditional Serbian alcoholic drink known as “Šljivovica” or “Prepečenica”, which is included in the UNESCO representative list of the intangible cultural heritage of humanity as an element of the intangible cultural heritage of Serbia (Source: UNESCO Bulletin, 2022) (Milošević T. et al., 2023). We have earlier described the properties of their trees and fruits (Milošević T., Milošević N., 2012; Milošević N. et al., 2017). Also, a great diversity of types belong to P. cerasifera Ehrh. (cherry plum), and P. spinosa L. (sloe) can be found in this country. Along this line, it can be said that Serbia is a very rich source of the biological diversity of plums.

The fruit of representatives of the Prunus genus consists of an epicarp (outer layer), a mesocarp (flesh), and an endocarp (stone). The stone of a plum consists of the seed covered with a hull. When used fresh or during processing, the flesh and skin of plums are the main raw materials, and the stones are generated as by-products. The plum seed or kernel may be a source of useful substances for food, cosmetics (e. g. personal care products), pharmaceutical industries (González-García et al., 2014; Plainfossé et al., 2019). Also, the seeds can be used to obtain generative rootstocks in horticultural practice and in breeding programs (Milošević T., Milošević N., 2018). The endocarp is the innermost layer of the pericarp, which directly surrounds the seeds. It may be very hard and nonedible as in drupes (also called stone fruits) such as members of the Prunus genus, i. e. plums, peaches, apricots and cherries (Carrillo-López, Yahia, 2019).

In pomological research, the stone of the Prunus genus represents a very stable feature of plum genotypes and serves for the determination and classification of cultivars (Behre, 1978; Woldring, 2000; Milošević T., Milošević N., 2018). However, during the last 50 years, with a few exceptions, in very complex botanical studies, stone dimensions, size and shape in the plum and other members are a crucial component for classification of systematic categories due to taxonomic complexity of the Prunus taxa within the Rosaceae family (Depypere et al., 2007, 2009; Burger et al., 2012; Bawari et al., 2022; Ropelewska, 2022). Behre (1978) reported that endocarp dimensions are very useful for the identification of P. domestica L., P. insititia L. and P. spinosa L. In general, reliable discriminating characters for species and subspecies of Prunus taxa identification are missing (Nielsen, Olrik, 2001).

The aim of this study was to characterize and evaluate the diversity of endocarps (stones) within and among traditional European and Damson autochthonous plums that are cultivated in Serbia and other western Balkan countries using essential morphological data (weight, dimensions, size, shape) in order to provide experimental evidence for the implementation of measures to safeguard this agricultural biodiversity. The secondary goal of this work was to reliably determine the degree of the possibility of identifying plum genotypes using the stone (endocarp) through multivariate statistical analysis.

## Materials and methods

Study area, plant material and measurements. Analysis was performed using a combination of weight, dimension and shape parameters of 5,500 endocarps (stones) belonging to two closely related Eurasian plum taxa (P. domestica L. and P. insititia L.). The analysis involved 55 genotypes i. e. accessions [43 accessions (78.18 %) belonging to P. domestica and 12 accessions (21.82 %) belonging to P. insititia]. Their name, series number and code were presented in Table 1.

**Table 1.**
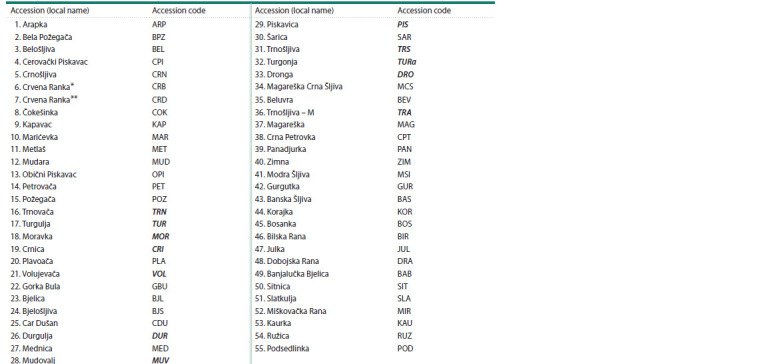
Name and code of autochthonous plum accessions Note. Accessions belonging to P. insititia L. are marked in bold-italic. * ‘Crvena Ranka’ var. ‘Bardaklija’ (P. domestica L.). ** ‘Crvena Ranka’ var. ‘Derosavka’ (P. domestica L.).

Ripe fruit and stone samples (25 fruits or stones in 4 replicates, n = 100 per one accession) were taken from a private orchard in the Prislonica village (43°33ʹ N, 16°21ʹ E) near Čačak city (western Serbia) established in 1998. Whole ripe fruits of each accession were harvested individually, manually and randomly in 2007. After this, the fruits were cut in half to extract the stones (endocarps). The removed stones were washed and cleaned of their flesh. After air-drying for 40 days at room temperature (20 °C in the shade), the undamaged and dry stones were placed in glass jars with hermetic closures and stored in a refrigerator at +4 °C. Stones (endocarps) were subjected to measuring in 2024.

**Fig. 1.**
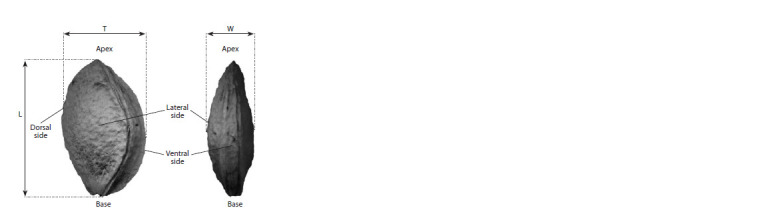
Overview of basic linear endocarp measurements Left: lateral view; right: ventral view (Van Zeist, Woldring, 2000).

The SW (g) was measured using MAULsteel 5000 G digital balance (Jakob Maul GmbH, Bad König, Germany). In order to determine the endocarp size, three major perpendicular dimensions i. e. L (mm), W (mm) and T (mm) were determined using a digital caliper Starrett 727 (Athol, NE, USA) with the accuracy of ±0.01 mm. The position of the measurements for L, T and W proposed by Van Zeist, Woldring (2000) was illustrated in Figure 1

Arithmetic mean diameter (Da, mm) and geometric mean diameter (Dg, mm) were computed from geometrical dimensions by Eq. (1) and Eq. (2) (Mohsenin, 1986):

**Formula. 1.**
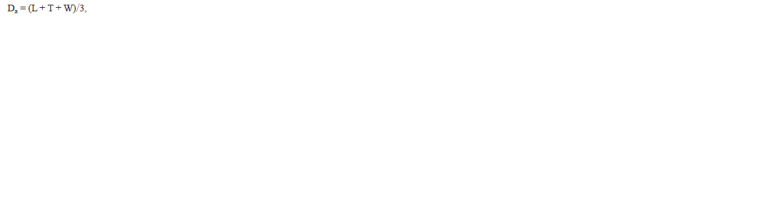
Formula. 1.

**Formula. 2.**
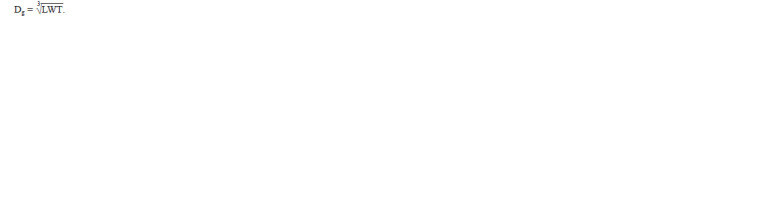
Formula. 2

The S (cm2) is a measure of the total area that the surface of the object occupies and was determined by approximating its shape to a sphere with the same geometric mean diameter by using Eq. (3) (Mohsenin, 1986):

**Formula. 3.**
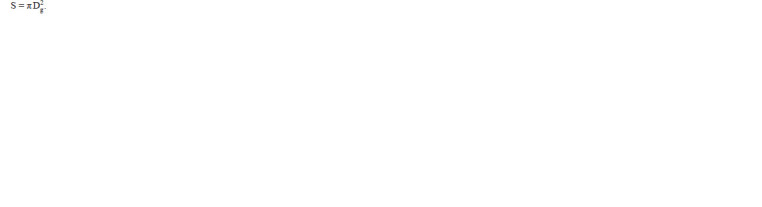
Formula. 3

The volume (V, mm3) of the endocarp was calculated by Eq. (4) (Mansouri et al., 2017), which is based on the assumption that plum endocarps are similar to a scalene ellipsoid where L > T > W (Munder et al., 2017):

**Formula. 4.**
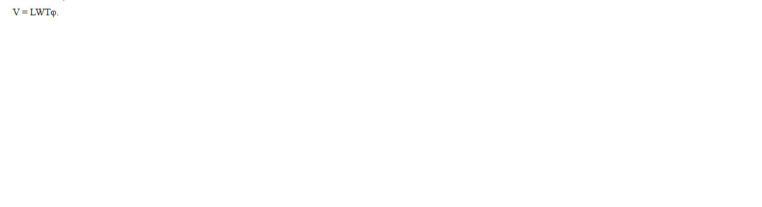
Formula. 4

Sphericity (φ) is defined as the ratio of the surface area of the sphere having an equivalent volume to that of the endocarp and the surface area of the endocarp. It is a measure of how spherical an object is. It was estimated based on the isoperimetric property of a sphere by Eq. (5) (de Figueiredo et al., 2011):

**Formula. 5.**
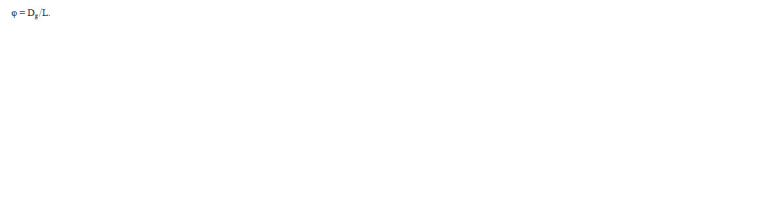
Formula. 5

Shape index (SI) and elongation ratio (E) were calculated using Eqs. (6) (Mohsenin, 1986) and (7) (Fıratlıgil-Durmuş et al., 2010):

**Formula. 6.**
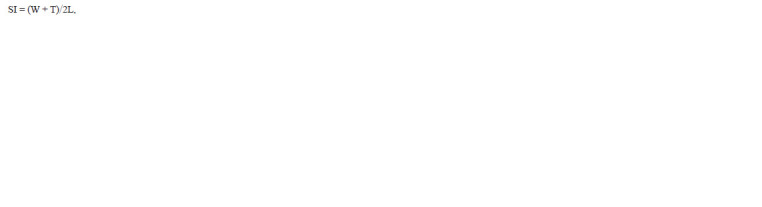
Formula. 6

**Formula. 7.**
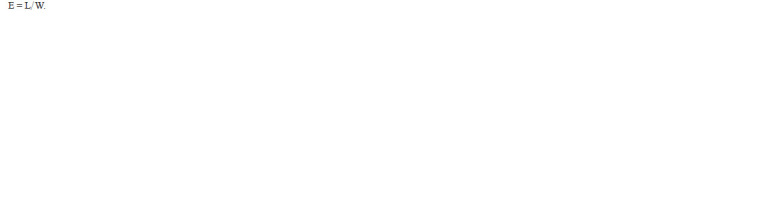
Formula. 7

Other indexes of endocarp shape were also calculated according to Behre (1978), Van Zeist, Woldring (2000) and Schmidt-Tauscher et al. (1996, cited in Pollmann et al., 2005). In order to have a more pronounced relationship between individual dimensions, the numbered values were multiplied by 100.

They can be represented as relative slenderness (RS) (Eq. (8)), roundness index (RO) (Eq. (9)) and Behre’s index (DE) (Eq. (10)) proposed by Behre (1978) and modified by Van Zeist, Woldring (2000):

**Formula. 8.**
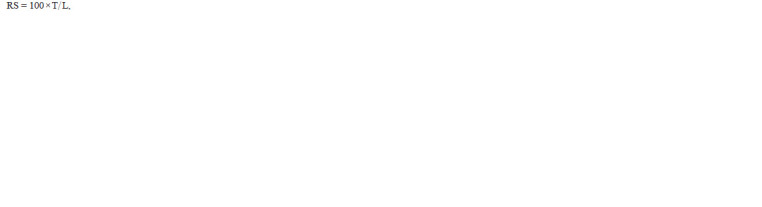
Formula. 8

**Formula. 9.**
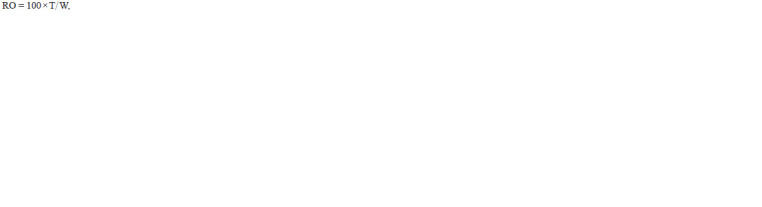
Formula. 9

**Formula. 10.**
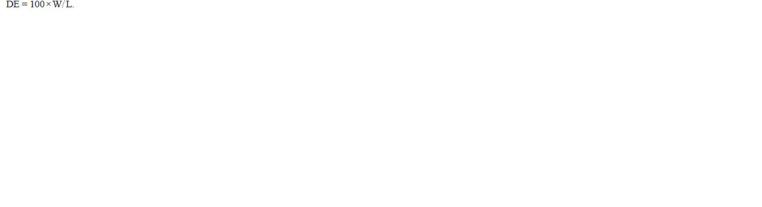
Formula. 10

Schmidt-Tauscher et al. (1996, cited in Pollmann et al., 2005) introduced a fourth index value which is calculated using Eq. (11). It was named Pollmann’s index (PI) because Pollmann et al. (2005) demonstrated its usefulness in differentiating the stones of modern plum cultivars:

**Formula. 11.**
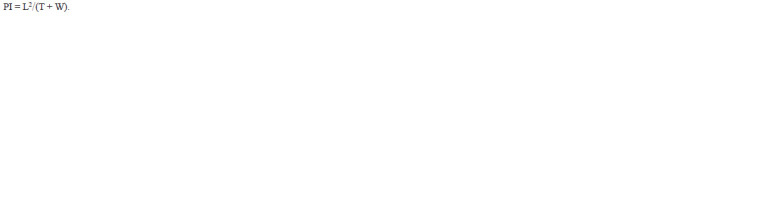
Formula. 11

Variation at different taxonomic levels was studied by analyzing the coefficients of variation (CV, %), which were interpreted following Rasch (1988, cited in Hübner and Wissemann, 2004), i. e. CV < 10 %, low variability; 10 % < CV < 20 %, normal variability; CV > 25 %, high variability of the character studied

Statistical analysis. Data were subjected to analysis of variance (ANOVA) using the Microsoft Office Excel software (Microsoft Corporation, Redmond, WA, USA) procedure followed by least significant difference (LSD) Fisher’s test at p ≤ 0.05 significance level. Pearson’s rank correlation matrix (α = 0.05) was performed using the R corrplot package version 4.0.2 (R Core Team, 2021). Principal components analysis (PCA) was performed and a biplot was designed using the XLSTAT v. 7.5 software package (Addinsoft, Paris, France).

## Results and discussion

Evaluation of endocarp dimensions and shapes

Data in Table 2 showed that SW significantly varied among accessions. High intra- and inter-variability between plum types was observed. The highest and statistically similar values were observed in ‘MUD’ and ‘CPT’ (both belonging to P. domestica), and the lowest, in ‘TRN’ and ‘CRI’ (both belonging to P. insititia). Twelve accessions (21.82 % of the total number) had SW > 1 g, whereas only four accessions (7.27 % of the total number) had SW < 0.5 g. The most numerous (70.91 %) were the accessions, the SW of which ranged between 0.5 and 1 g.

**Table 2.**
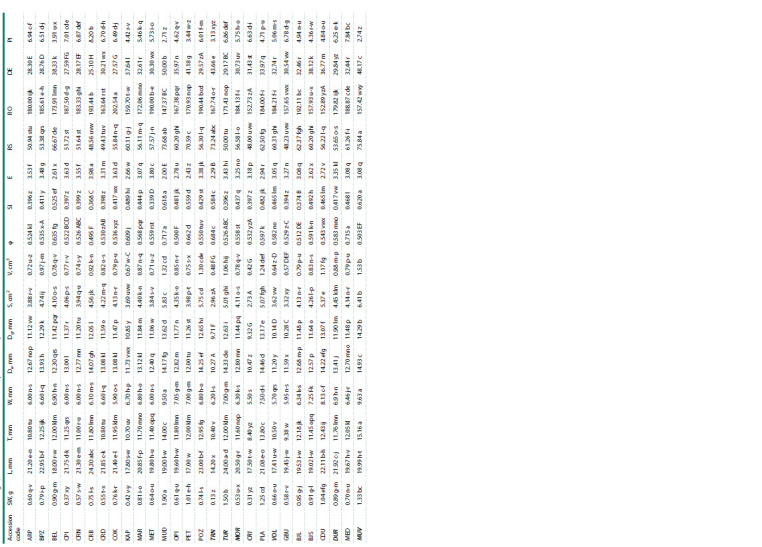
Stone weight, stone dimensions and size and shape indexes of plum accessions

**Table 2.end:**
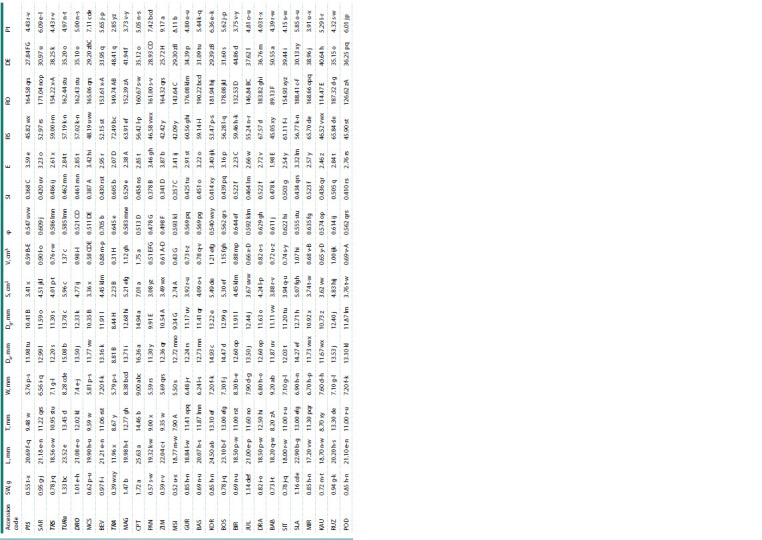
Table 2.end Note. Codes of members of P. insititia L. are marked in bold-italic. Mean values with different letters in a column differ significantly at p ≤ 0.05 by LSD test. Means are initially designated by small letters and afterwards by capital letters due to great variation in means.

It is known that the former Yugoslavia, i. e. the Western Balkan region, is a very rich source of plum germplasm, especially that of European and Damson plums, cherry plums and sloes (Milošević T., Milošević N., 2012). Considering this, a large number of researchers highlight data related to their biological, agronomic and pomological characteristics (Milošević T. et al., 2023). Thus, Milošević N. et al. (2017) and Glišić et al. (2023) reported SW between 0.57 and 1.49 g or 0.57 and 2.39 g, respectively, for local genotypes belonging to European plum in the Čačak area (western Serbia). Drkenda, Kurtović (2012) reported values between 0.84 and 1.21 g for six local cultivars from Bosnia and Herzegovina. For accessions belonging to European and Damson plums from Montenegro, Jaćimović et al. (2011) and Šebek (2013) reported that SW varied from 0.46 to 2.20 g and 0.16 to 2.20 g, respectively. For nine domestic and local plums grown in Turkey, Gunes (2003) noted SW from 0.26 to 0.99 g. Our data for SW are within the limits of the results of the mentioned authors. However, in taxonomic description and morphometric analysis, SW is not a reliable indicator for the determination, systematization and segregation of members of the Prunus genus (Depypere et al., 2007; Mijnsbrugge et al., 2013; Sarigu et al., 2017; Heidari et al., 2022; Kosina, 2023) due to the negative “effect of controlled moisture” (Sheikh et al., 2021) and state of the seed (embryo or kernel) inside the endocarp (das Graças Souza et al., 2016; Sheikh et al., 2021). All of the above authors favored dimensional measurements of the endocarps.

The ANOVA showed signifant differences among the ascessions for endocarp L, T and W (Table 2). These data are in agreement with the results of Woldring (2000), Van Zeist, Woldring (2000), Depypere et al. (2007) and Heidari et al. (2022) who reported that all three linear dimensions of endocarps significantly varied among plum genotypes. The highest endocarp L was observed in ‘CPT’, ‘KOR’, ‘CRB’ (all belonging to European plum), ‘TUR’ and ‘TURa’ (both Damson plums) with no significant differences among them, whereas the lowest L was found in ‘TRA’ (Damson plum). Out of the total number of accessions, 50.91 % had L > 20 mm.

Regarding endocarp T, ‘MUV’ had the highest value, whereas the lowest was found in ‘MSI’. In the case of endocarp W, a very high number of the largest and/or smallest values overlapped. The highest values were observed in the set of accessions such as ‘MUV’, ‘MUD’, ‘BAB’ and ‘CPT’ with no significant differences among them. In contrast, the lowest and similar values were found in 17 accessions (30.91 %) i. e. ‘ARP’, ‘CPI’, ‘CRN’, ‘COK’, ‘MET’, ‘TRN’, ‘CRI’, ‘VOL’, ‘GBU’, ‘BJL’, ‘PIS’, ‘MCS’, ‘TRA’, ‘PAN’, ‘ZIM’, ‘MSI’ and ‘BAS’. Some of them, such as ‘ARP’, ‘CPI’, ‘CRN’ and ‘MET’, had identical mean W values.

Taking into account the absolute values of the three linear dimensions, the descending order was L > T > W, which is in accordance with the recommendations of morphometric analysis of plum endocarp proposed by Behre (1978) and Woldring (2000). Also, the values of endocarp dimensions obtained in our study were within the limits described by Van Zeist, Woldring (2000).

Following the procedure proposed by Caillavet and Souty (1950), values of all three dimensions (L, T and W) were transformed into the parameter denominated “size” or Da and/ or Dg. In the present study (Table 2), both Da and Dg values significantly varied among accessions.

A similar finding applies to endocarp S and V, respectively. The lowest value for all four parameters was found at ‘TRA’ belonging to P. insititia. The highest endocarp “size”, calculated as Da and Dg, S and V, was observed in ‘CPT’ belonging to European plum. ANOVA results showed that the differences among accessions for these properties were clear and significant. Only two accessions (3.64 %) had endocarp S between 6 and 7 cm2, whereas nine accessions (16.36 %) had S between 5 and 6 cm2. Other accessions (80 %) had S < 5 cm2. Otherwise, the knowledge of a specific surface area (S) could be a relevant tool to determine the shape. Other authors also report large variations in the endocarp size, S and V in plums (Sheikh et al., 2021), cherries (Pérez-Sánchez et al., 2010; Ganopoulos et al., 2015; Khadivi et al., 2022), peaches (das Graças Souza et al., 2016) and apricots (Gezer et al., 2002).

The fruit or stone (endocarp) shape is determined in terms of its φ. Moreover, φ is an expression of the shape of a solid related to that of a sphere of the same volume. In the current study, ‘MUD’ and ‘MED’ had similar and the highest φ values, whereas the lowest was observed in ‘PAN’ (Table 2).

In general, φ is used for determining the similarity of a fruit or a stone to a sphere. Hence, higher values of φ indicate the tendency of endocarps towards sphericity (Sheikh et al., 2021). The φ value more than 0.7 i. e. 70 % is assumed to be spherical (Garnayak et al., 2008). However, average φ values for accessions were much lower than 0.7 or 70 % with the exception of ‘MUD’, ‘MED’ and ‘BEV’.

The shape parameters such as SI and E indicate the shape tendency of the endocarps. Both indexes in the present study showed high variability among accessions (Table 2). ‘MUV’ and ‘MUD’ had the highest and similar SI values whereas the lowest were observed in ‘MET’. The highest E value was in ‘CRB’, and the lowest, in ‘BAB’. Lower values of these shape parameters indicate the tendency of endocarps to being flat and oblong in shape as previously reported by Sheikh et al. (2021) for plum kernels. Based on the values of the above endocarp shape indexes in this study and their comparison with the IBPGR recommendations on stone shapes (lateral view) (Cobianchi, Watkins, 1984), it can be said that elongated and ovate shapes dominate, and sporadically, rounded ones appear.

Endocarp shape indexes proposed by Behre (1978), Van Zeist, Woldring (2000) and Pollmann et al. (2005) of the evaluated plum accessions were presented in Table 2.

The ANOVA showed significant differences among accessions for all four indexes. The highest RS index (endocarps in lateral view) denominated as relative slenderness was found in ‘MUD’, ‘MUV’ and ‘TRN’ with no significant differences between them. The lowest and statistically similar values of this index were discovered in ‘ZIM’ and ‘MSI’, both belonging to P. domestica. Our values of this index for 45 accessions (81.82 %) were within the limits described previously (Van Zeist, Woldring, 2000; Pollmann et al., 2005), while 10 accessions (18.18 %) had slightly lower values than the minimum described by the above authors. Otherwise, the more slender the stone, the lower the index value (Depypere et al., 2007). The high variability of this index was previously described by Van Zeist, Woldring (2000).

With regard to RO index, which expresses the roundness of the endocarp in apical view, the highest value was observed in ‘COK’, and the lowest, in ‘BAB’, with 2.27-fold difference between them. The minimum and maximum values according to the descriptors proposed by Van Zeist, Woldring (2000) and Pollmann et al. (2005) for this index are 112.15 and 225.45, respectively, which was the case in our study with the exception of ‘BAB’ that had a much lower value than the minimum limit. In general, endocarps with strongly domed sides show a low RO value, while in rather flat stones, this value is relatively high and always more than 100 (Van Zeist, Woldring, 2000).

Similarly to previous indexes, DE index, which represents endocarps in ventral view, varied among and within accessions. It was the highest in ‘BAB’, and the lowest and statistically similar one was observed in ‘ZIM’ and ‘CRB’, all belonging to P. domestica. Our values were generally closer to the minimum values proposed by Van Zeist, Woldring (2000), which varied from 26.30 to 106.32.

PI significantly varied among and within accessions, ‘ZIM’ being the accession with the highest value, whereas the smallest value was found in ‘MUD’ with 3.38-fold difference between them. According to Pollmann et al. (2005), minimal and maximal values of this index ranged between 1.27 and 7.68, which was confirmed by our data. Depypere et al. (2007) reported that the PI index value was highly variable for P. domestica and P. insititia.

Evaluation of variability and mean values of properties between plum types

With regard to the variability of mean values of properties evaluated by means of the coefficients of variability (CV, %), the results showed that L, Da, Dg and somewhat φ had a low variability (CV < 10 %) in P. domestica, and only φ and RO, in P. insititia (Table 3).

**Table 3.**
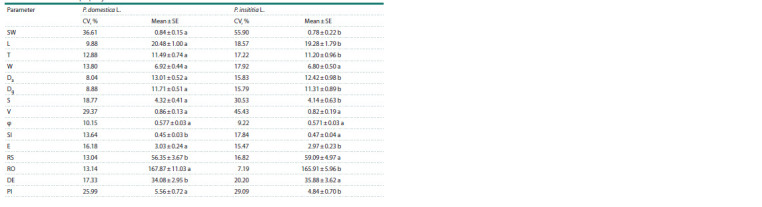
Intraspecific variability for P. domestica and P. insititia expressed by means of the coefficients of variability (CV, %) and mean values for each property evaluated Note. CV > 25 % indicates high variability; 10 % < CV < 20 % indicates normal variability; CV < 10 % indicates low variability (following Rasch, 1988; cited in Hübner, Wissemann, 2004). Mean values with different letters in a row differ significantly at p ≤ 0.05 by LSD test

Parameters T, W, S, SI, E, RS, RO and DE in P. domestica and L, T, W, Da, Dg, SI, E, RS and DE in P. insititia had a lowto- normal variability (10 % < CV < 20 %). Parameters SW, V and PI in both European and Damson plums, and S and DE in P. insititia were moderate to highly variable (CV > 25 %). It appears that the mean value of the coefficients of variation of all morphological levels for accessions belonging to P. domestica was significantly smaller (CV = 16.51 %) compared to the accessions belonging to P. insititia (CV = 22.20 %) (data not shown). Our results were in good agreement with the results found by Depypere et al. (2007) for several indexes such as W, T, DE, RS and PI.

Regarding mean values of properties evaluated for both European and Damson plums, the results from Table 2 showed that there were significant differences between them with the exception of W, V and φ. These three values were statistically similar for both plum types. Accessions belonging to P. domestica had higher mean values for SW, L, T, Da, Dg, S, E, RO and PI than accessions belonging P. insititia. On the contrary, accessions belonging to P. insititia had higher SI, RS and DE mean values than representatives of European plum.

Correlations among variables and principal component analysis (PCA)

**Fig. 2.**
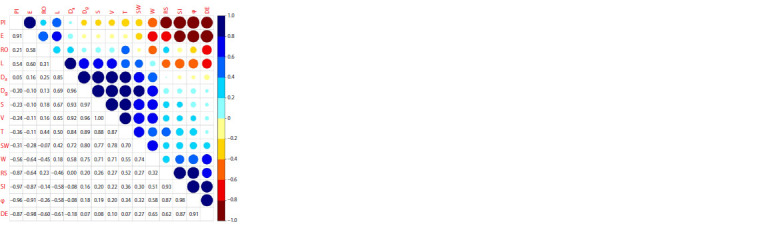
Correlation matrix of Pearson’s correlation coefficients (r) between the mean values of the endocarp parameters evaluated.

Relationships among 15 endocarp parameters were studied and Pearson’s correlations were calculated and were presented graphically (Fig. 2). Significant correlations were found among most of the studied traits, but high values were noted only in some cases

SW was significantly correlated with all parameters and indexes with the exception of RS and RO, indicating that accessions with a big stone tend to have greater endocarp dimensions and higher indexes values in general and vice versa. Hence, all parameters can be used to predict each other. Similar tendencies were observed in cherry plum (Heidari et al., 2022). However, the intensity of correlations between some parameters differed. Namely, strong positive correlations were observed between SW and T, W, Da, Dg, S and V, whereas other correlations were weak, which shows that endocarps have some very similar properties and that their values are not greatly influenced by genotype. In addition, SW was negatively correlated with E and PI. These findings are in good agreement with the results obtained on hazelnut (Milošević T., Milošević N., 2017).

L, W and T were significantly correlated with endocarp indexes with the exception of L vs W, T vs E and/or DE. The absence of significant correlation between L and W is in agreement with the results of Kosina (2023) for P. spinosa. So, these traits were considered to be independent. Strong correlations were observed between L and Da and between T and Da, Dg, S and V, indicating that endocarps with higher L and T tend to have a greater endocarp size. Both Da and Dg showed very strong mutual correlation, and also with endocarp S and V. However, relationships of Da and Dg with other indexes were small and not significant. φ was strongly positively correlated with SI, RS and DE, but strongly negatively correlated with E and PI. Both S and V were significantly correlated with SW, endocarp dimensions and size parameters, whereas correlations with all indexes were minor and not significant.

There was an extremely strong mutual correlation between S and V indicating that an endocarp with higher S values tends to have higher V values and vice versa. On the other hand, there were no significant correlations between V and all shape indexes. SI was strongly negatively correlated with E and PI, and positively with RS and DE. Similar trends have been reported for European plum kernels (Sheikh et al., 2021). E vs RS and DE were negatively correlated, and positively correlated with RO and PI. RS showed negative correlations with PI, and positive with DE, whereas both RO and PI were negatively correlated with DE.

**Fig. 3.**
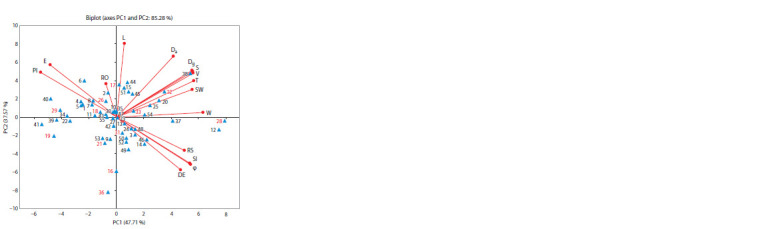
Segregation of European and Damson plum accessions according to endocarp properties (linear dimensions, size and shapes) and their projections on the first (PC1) and second factors (PC2) of the component analysis. See Table 1 for accession series numbers and accession codes; the series number in red represents accessions belonging to Damson plum.

Principal component analysis (PCA), as a statistical tool, is performed to reduce the number of effective traits and to identify groups. In the current study, using the 15 analyzed parameters, the first three principal components accounted for 96.67 % of the total variance. PC1 explained 47.71 % of the total variation, while PC2 explained 37.57 % and PC3 explained 11.39 % (Fig. 3). According to the correspondence between the PCA and the original properties and eigenvectors, SW, T, Dg, φ, S, V, and SI made the largest contributions to PC1 with positive values, while PI had a negative contribution. As a result, genotypes such as ‘MUD’, ‘PLA’, ‘CDU’, ‘MUV’, ‘TURa’, ‘DRO’, ‘MAG’, ‘CPT’ and ‘RUZ’ tended to exhibit higher SW, T, Dg, φ, S, V, and SI values but lower PI values. In contrast, accessions like ‘ARP’, ‘CPI’, ‘CRN’, ‘CRD’, ‘MET’, ‘MOR’, ‘CRI’, ‘GBU’, ‘PIS’, ‘MCS’, ‘PAN’, ‘ZIM’ and ‘MSI’ displayed the opposite trend.

For PC2, positive values were associated with L, Da, and E, whereas DE contributed negatively. This suggests that genotypes like ‘BPZ’, ‘CRB’, ‘COK’, ‘MAR’, ‘POZ’, ‘TUR’, ‘DUR’, ‘KOR’, ‘BOS’, and ‘SLA’ exhibited higher L, Da, and E values, while accessions such as ‘BEL’, ‘KAP’, ‘OPI’, ‘PET’, ‘TRN’, ‘VOL’, ‘BJS’, ‘TRS’, ‘TRA’, ‘GUR’, ‘BIR’, ‘SIT’ and ‘MIR’ showed lower values of these parameters.

Finally, RS and RO contributed to the positive values of PC3, whereas W contributed negatively, indicating that genotypes such as ‘BJL’, ‘MED’, ‘BAS’, and ‘DRA’ were predisposed to higher RS and RO values, while accessions like ‘SAR’, ‘BEV’, ‘JUL’, ‘BAB’, ‘KAU’, and ‘POD’ tended to have lower W values

## Conclusion

The stones of accessions belonging to P. domestica L. and P. insititia L. showed characteristic differences in size and shape features, which greatly facilitate the identification of genotypes or accessions. Each of them could be identified by means of dimensions and morphological features of the endocarps. In the present study, most endocarp parameters were found to be very useful for further taxonomic research, based on their low variability in both P. domestica and P. insititia. However, some parameters such as SW, V and PI exhibited a high variability and we suggest omitting their use for taxonomic purposes in some cases or for them to be used in a limited way. In general, the examined parameters varied less in accessions belonging to European plum compared to Damson plum genotypes. In addition, the mean values of SW, L, T, Da, Dg, S, E, RO and PI were higher in P. domestica type compared to P. insititia, while the mean values of W, V and φ were similar. Others, such as SI, RS and DE were higher in Damson plum. However, due to overlapping ranges in most cases within and between plum types and accessions, the use of one or two endocarp parameters is not satisfactory for discrimination between Eurasian plum taxa. The multivariate analysis as a statistical tool can be useful for higher quality dispersion, segregation and determination of plum accessions, but in these analyses, the overlapping of values of endocarp morphological parameters also occurs. Finally, based on our results obtained on dry endocarps and the results of other researchers who experimented with fresh stones, we recommend full hydration of dried endocarps, as this restores the original dimensions and shape of the sampled endocarps

## Conflict of interest

The authors declare no conflict of interest.
